# Case of an Imperforate Rectum

**Published:** 1791

**Authors:** Edward Ford

**Affiliations:** Surgeon of the Westminster General Dispensary.


### X. *Case of an Imperforate Rectum. By the same.*

MARCH 6th, 1791, I was desired to see a male infant, two days old, who was supposed to have an imperforate rectum. He appeared to be a strong healthy child, well formed in every other respect, had taken nourishment the day before, and as he exhibited externally no marks of mal-conformation, when examined at his birth, it was not supposed that he laboured under this defect till it was found that no evacuation had taken place through the intestines, that he rejected his food, and vomited up every thing he had taken.

When I saw the child he was continually vomiting; the matter thrown up was of a dark yellow colour, and fœtid, and the abdomen was tense and swelled: in other respects he looked healthy, had voided his urine properly, and the anus was naturally formed as far as regarded its external appearance.

I endeavoured to introduce my little finger through the sphincter ani into the rectum, but found an uncommon resistance in the first attempt, the parts not admitting of being dilated as usual; and when this difficulty was with some force force overcome, at the diftance of an inch from the external parts, there was an obftruction to be felt, which refifted every effort I made to penetrate it, firft with the nail of my finger, and afterwards with the blunt end of a probe.

The firft confideration which offered to my mind, was to perforate the obftruction with a fmall trocar; and in order to do this as fafely as poffible, a fmall catheter was introduced through the urethra into the bladder, which ferved as a direction to avoid wounding thofe parts in the operation. The canula of the trocar was then introduced into the anus, under my finger, which defended the urethra, and was fixed as well as I could againft the obftructed part of the canal.

The ftilet was then carried up through the canula, and pufhed through the obftruction in a direction rather backwards towards the os facrum. On withdrawing the ftilet it was followed by a difcharge of fæces, through the canula, which continued for an hour fo as to form rather a copious ftool. Upon taking out the canula, a bougie was attempted to be introduced through the artificial opening, but without effect.

The child was now left an hour, and on my return I found his belly more tenſe, and that his vomiting continued. I therefore directed ſeveral clyſters of oil and water to be thrown up by means of a ſmall pipe which was fortunately conveyed through the artificial opening into the gut. Theſe clyſters brought off a conſiderable quantity of fæces, but did not ſeem thoroughly to empty the inteſtinal canal; ſo that I deemed it expedient to attempt an enlargement of the opening, by means of the point of a blunt gorget carried up in the groove of a common director. A farther diſcharge of fæces enſued; and the child was then put into a warm bath, and caſtor oil was afterwards adminiſtered by the mouth. Nothwithſtanding theſe remedies, the vomiting continued, the child became convulſed, and died in the courſe of the following night.

Upon opening the body the next morning, I found marks of conſiderable inflammation in the inteſtines, principally in the larger ones, which were inflated to a great degree. There was no obſtruction, however, to be found in any part of the inteſtinal canal except that in the rectum.

The drawing which accompanies this paper will ſhow the manner in which the inteſtine terminated terminated in a blind pouch, at the diſtance of an inch from the anus, in the hollow of the os ſacrum*. The ſpace between the inteſtine and the anus was lined with an inelaſtic ligamentous ſubſtance, which would probably have produced much inconvenience to the patient in retaining his ſtools, had the operation performed protracted his exiſtence.

*Golden Square,*
May 20, 1791.

